# A Submerged Optical Fiber Ultrasonic Sensor Using Matched Fiber Bragg Gratings

**DOI:** 10.3390/s18061942

**Published:** 2018-06-14

**Authors:** Xiaohong Bai, Manli Hu, Tingting Gang, Qiangzhou Rong

**Affiliations:** School of Physics, Northwest University, Xi’an 710127, China; baixiaohonglxl@163.com (X.B.); tingtinggang1@163.com (T.G.)

**Keywords:** fiber optic ultrasonic sensor, matched fiber Bragg grating, sinusoidal ultrasound

## Abstract

A novel kind of fiber optic ultrasonic sensor based on matching fiber Bragg gratings (FBGs) is proposed and demonstrated. The sensors consist of a pair of matching FBGs fixed to a special bracket. The bracket plays a role in stretching and squeezing the FBGs, with the push–pull effect efficiently coupling the ultrasonic signal to the sensor, thus, improving the sensor’s sensitivity. Side-band filtering technology-based intensity interrogation was used to detect ultrasounds in water. With the synergic effect of the matching FBGs, the sensor performed with a high signal-to-noise ratio (56.9 dB at 300 KHz, 53 dB at 1 MHz and 31.8 dB at 5 MHz) and the observed ultrasonic sinusoidal signals were undistorted and distinguishable in the time domain.

## 1. Introduction

Maritime security is a very important topic within sea transportation, submerged navigation, and marine disaster prediction. To reduce marine accidents, maritime security technology for underwater target detection is required [1]. Previous methods for marine target monitoring (MTM) have mainly relied on satellites, space-based radar, and visual searching. While all of these are valid approaches, they cannot be used for underwater target detection [2,3,4]. In comparison, acoustic sensors have presented outstanding performances in MTM and have attracted a great deal of attention from scientists.

Thus far, the traditional piezoelectric transducer (PZT) has principally been used for ultrasonic detection. PZTs respond to an ultrasonic wave (UW) by creating an electrical signal via the piezoelectric effect. This method has some inherent disadvantages, including: Its bandwidth is narrow, owing to strong resonant effects; it is susceptible to electromagnetic disturbances; its spatial resolution is limited due to its large size; and it is only suitable for single-point detection, which leads to poor multiplexing ability [5,6].

Although some researchers have tried to replace PZT with other electrical methods to solve these problems, their efforts have not proved effective. In 1966, Kao and Hockham reported on optical fiber communication and studied the modulation of light transmission in optical fibers, which was the prelude to the emergence of the optical fiber sensor [7]. Compared with conventional ultrasonic sensors, the advantages of fiber optic-based ultrasonic sensors far outweigh their disadvantages. Not only are they suitable for broadband weak signal detection and long-term use in especially harsh environments, but they can realize the goal of networked detection and, thus, improve detection efficiency [8,9]. This means that research on fiber optic-based ultrasonic sensors is significant in many fields.

The fiber optic-based ultrasonic sensors detect UWs through high-speed recording of the intensity, wavelength, phase, and polarization of light propagating through optical fibers. Previously reported fiber optic-based ultrasonic sensors mainly include intensity modulation of fiber optic sensors (IMFOS), interferometric fiber optic sensors (IFOS), and fiber optic grating sensors (FOGS) [10]. Although IMFOS have shown high sensitivity and broadband frequency response in the detection of UW, they still have some shortcomings, such as poor stability of the UW detection and low signal-to-noise ratio (SNR) and single-point detection [11,12].

By contrast, IFOS are more compact, especially those based on the Fabry–Perot interferometer (FPI). They have wide bandwidths and multiple methods of demodulation. However, inevitably, IFOS are also sensitive to other physical variables (for example, temperature and low frequency strain) and the robustness of the sensors is poor, which makes them vulnerable to damage. Hence, it is necessary for IFOS to incorporate other technology in the demodulation system to improve SNR. Multiplexing is also a difficult problem to solve [13,14].

In comparison, FOGS have many merits, including: Their wavelength is an absolute parameter, with the measurement results not being affected by system fluctuations and they cope well with interference. Furthermore, the reflected wavelength of the fiber Bragg grating (FBG) has a narrow bandwidth. Moreover, compared to the multiplexing system based on IFOS, it is more simplified and the multiplexing is improved [15,16].

Presently, FBGs mainly use silica optical fibers (SOF) and polymer optical fibers (POF). Stefani et al. investigated the behavior, under dynamic excitation, of polymer fibers made of polymethyl methacrylate (PMMA). The results show that the Young’s modulus of POF is much less than SOF, making it a good candidate for optical fiber sensors [17]. However, even though the properties of POF show some advantages compared with SOF and some applications have been proposed for polymer FBG sensors, the technology for writing the Bragg grating in POF is still under development and there are difficulties related to coupling POF with SOF [18,19]. FBGs based on SOF are, therefore, still the primary method used for ultrasonic measurement [20].

In this paper, we propose a novel fiber optic sensor based on matching FBGs and apply it to acoustic measurements. As a universal concept, “matching” is often used in many fields, for example, demodulation techniques, chirped-pulse-amplification systems, and sensor networks [21,22,23]. The novelty of this article is the use of the concept of matching FBGs in an ultrasonic sensor. Additionally, compared to other fiber optic ultrasonic sensors, it has excellent ultrasonic sensitivity and the ultrasonic sinusoidal signals we detected were undistorted and distinguishable in the time domain.

## 2. Sensor Fabrication and Principles

**Figure 1:**
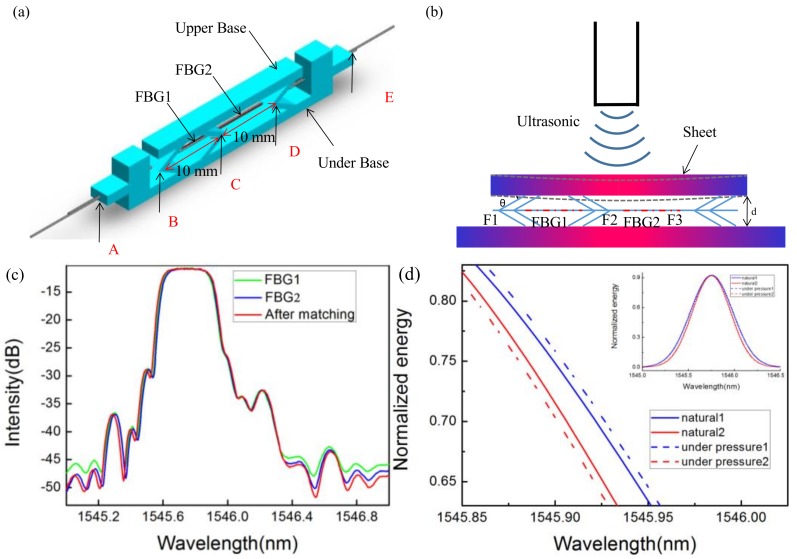
(**a**) A schematic diagram of the sensor; (**b**) force analysis of the sheet with the applied ultrasonic wave; (**c**) the reflected spectrum of the FBGs; and (**d**) the wavelength shift calculated by using the theory of fiber grating sensors.

The structure schematic diagram of the sensor probe is shown in Figure 1a. The upper base, a bracket made of polymethyl methacrylate (PMMA), was used as a transducer for transferring the UW to the upper base strain. The reason we chose PMMA to sense UWs is that it has the properties of acid–alkaline resistance, a long lifespan, good insulation, and low weight. Compared to other materials, the Young’s modulus of PMMA is very small (3 GPa), so it is sensitive to weak vibrations. Theoretically, the properties of the FBG in POF are better than in SOF. However, in practice, we need to consider all steps of the process, including writing the Bragg grating and issues of connecting and coupling. We chose the FBGs in SOF made by Beijing Xizhuo InfowareLab Information Technologies, Inc. The length of the grid region is an important influence on the sensor performance; according to the research of the FBG in the sensor area, we chose FBGs of the length 10 mm [24].

Two FBGs were passed through a hole (of the diameter 0.5 mm) drilled in the bracket. One FBG was fixed between points B and C (see Figure 1a), while the other was fixed between points C and D using a cyanoacrylate adhesive. The line segment, BD, is 20 mm long and has C as its midpoint. In our work, both FBGs were etched to nearly 35 μm in diameter, with the aim to improve sensitivity to the strain caused by upper base vibrations. We found that further etch of the FBGs caused them to break easily. During the pretreatment process, pre-stress functions maintained the extension of the FBGs to ensure wavelength matching and keep them tightened. Additionally, points A and E were fixed to prevent the pre-stress from weakening and to enhance the structural stability.

The green, blue, and red lines in Figure 1c show the reflectance spectra of FBG1, FBG2, and after matching, respectively. The reflection spectrum functions of FBG1 and FBG2 are assumed to be $R_{0}\left(\lambda \right)$ and R(λ), respectively. The match reflection spectrum function is $R_{0}\left(\lambda \right)\times R\left(\lambda \right)$. Since the reflected structure, $R_{0}\left(\lambda \right)$, and R(λ), show nearly a Gaussian distribution, they can be expressed as:

(1) $$R_{0}\left(\lambda \right)=R_{S}\cdot \exp\left[-4\cdot \ln2\cdot \frac{{\left(\lambda -\lambda_{S}\right)}^{2}}{W_{S}^{2}}\right]$$

(2) $$R\left(\lambda \right)=R_{B}\cdot \exp\left[-4\cdot \ln2\cdot \frac{{\left(\lambda -\lambda_{B}\right)}^{2}}{W_{B}^{2}}\right]$$

where *λ*_S_, *λ*_B_, *R*_S_, *R*_B_, *W*_S_, and *W*_B_ are the center wavelength, peak reflectivity, and full width at half maximum (FWHM) of FBG1 and FBG2, respectively. Then, the spectrum function, inputted to the photodetector, can be expressed as:

(3) $$I\left(\lambda \right)=\frac{\alpha I_{0}}{4}\cdot R_{0}\left(\lambda \right)*R\left(\lambda \right)$$

where α is the coupling loss; $I_{0}$ is the input intensity, and * is the convolution. Thus, the optical power received by the photodetector is:

(4) $$P_{D}\left(\lambda \right)=\int_{-\infty }^{+\infty }I\left(\lambda \right)d\lambda$$

The maximum output power occurs when the center wavelength of FBG1 matches the center wavelength of FBG2 [25].

In our system, there are two main reasons for a wavelength shift under the UW. Firstly, as shown in Figure 1b, the sheet will deform when the ultrasound is applied to its surface. Additionally, the displacement of the sheet’s center point can be expressed, according to the equations of elasticity:

(5) $$f=\frac{k\cdot P\cdot \left(1-\upsilon^{2}\right)}{\pi^{4}abEt^{3}}$$

where *t*, *a*, *b*, *E*, and υ are the thickness of 2 mm, length of 34 mm, width of 4 mm, Young’s modulus of 4.19 GPa, and Poisson ratio of the sheet of 0.39, respectively. *P* is the sound pressure and *k* is a dimensionless constant. The bending deformation was calculated to be 4.35 × 10^−8^ m under 1 Pa of sound pressure. According to the geometric relationship between the sheet and the holder, the deformation of the FBG can be derived as:

(6) $$∆L=\frac{\sqrt{3d^{2}+2df+f^{2}}}{2}-d\cos\theta$$

where *d* is the width of the holder and *θ* = 30° is the angle between the sheet and the holder. Thus, the strain of the FBG was 4.53 × 10^−6^. In addition, FBG1 is stretched, while FBG2 is squeezed because of their structures. Thus, as shown in Figure 1d, the center wavelength of FBG1 shows a bathochromic effect, while the center wavelength of FBG2 shows a hypsochromic shift under 1 Pa of sound pressure. The change in energy caused by the wavelength mismatch is detected by the sensor system. Theoretically, the synergic effect of the matching FBGs can be observed when they are used for ultrasonic detection, as the matching of them shows a larger effect than when each is used individually.

The UW also acts directly on the grating through the water, which is the second reason for the wavelength shift. To simplify the analysis, we consider the UW to be a pure transverse stress wave, $\sigma_{z}=\nu \left(\sigma_{x}+\sigma_{y}\right)=0$, that changes with time, according to a cosine curve. The axis stresses are then:

(7) $$\sigma_{x}=-\sigma_{y}=P\left(x,t\right)=P_{0}\cos\left[2\pi f\left(t-x/\nu_{m}\right)\right]$$

where *ν_m_* is the ultrasonic velocity and *P*_0_ is the ultrasonic stress amplitude. There are two orthogonal polarization modes when the FBGs are forced by the transverse stress wave. We obtain the following equations, according to fiber grating theory:

$$∆\lambda_{x}=-∆\lambda_{y}=-\frac{n_{eff}^{3}}{E}\left[\left(1+\nu \right)\left(p_{11}-p_{12}\right)\right]\sigma_{x}$$

(8) $$\frac{∆\lambda_{x}}{P_{0}}=-\frac{n_{eff}^{3}\Lambda }{E}\left[\left(1+\nu \right)\left(p_{11}-p_{12}\right)\right]$$

where *n_eff_* and *E* are the effective refractive index and the period of the FBG, $p_{11}=0.121$, and $p_{12}=0.27$. From the equations, we can see that there is birefringence under the transverse ultrasound [26], which causes the wavelength at the center of the FBGs to drift and the sensitivity to increase. Given the above, the causes of the wavelength shift are both immediate and remote. The combination of the two functions means the sensor performs with a high sensitivity.

## 3. Experimental Results and Discussion

**Figure 2:**
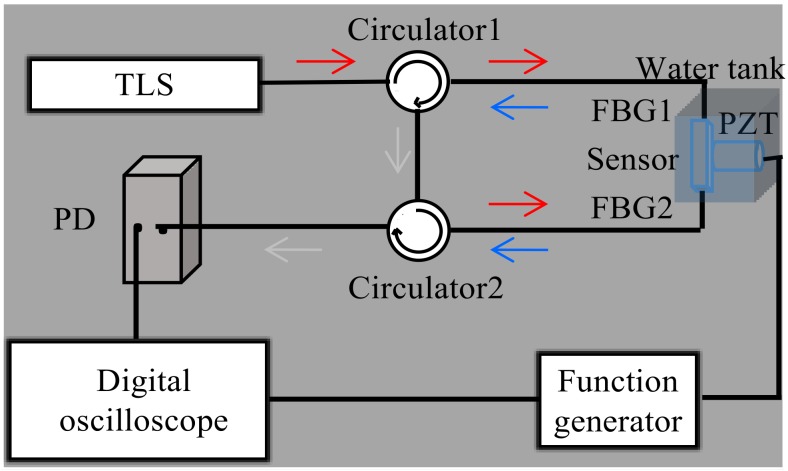
Schematic diagram of the ultrasonic detection system.

A schematic of the ultrasonic detection system is shown in Figure 2. The sensor and PZT were placed in a transparent water tank (20 × 20 × 20 cm) to reduce ultrasonic loss. In the set, the light, from a tunable laser (Santec-710, Santec, Toyota, Japan) with a 100 KHz line width and a 0.1 pm tunable resolution, was shone into FBG1 through Circulator 1, and the reflected light was imported into Circulator 2. The light then entered FBG2 and the resulting reflection was transmitted into a photodiode (PD, New Focus, Shanghai, China) with a bandwidth of 10 MHz at a gain of 0 dBm. At this point, the central wavelength of FBG1 matches the central wavelength of FBG2, which gives rise to the maximum energy output. As described above, the central wavelength will mismatch when the UW is applied to the sensor. Using the spectral side-band filtering technique, the ultrasonic responses of the sensor could be obtained. Additionally, we employed a piezoelectric transducer (PZT, mono-crystalline longitudinal wave probe) driven by a function generator as a source for UWs with frequencies of 300 KHz, 1 MHz, and 5 MHz.

**Figure 3:**
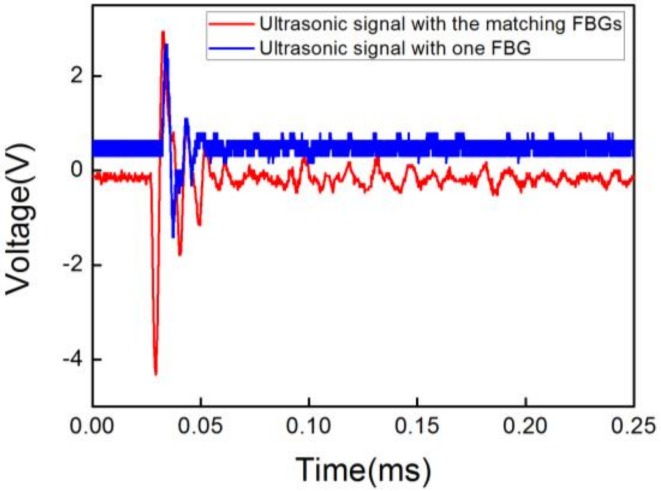
Responses of the sensor based on matching FBGs and one FBG to a one-cycle ultrasonic pulse.

To provide a contrast in the analysis, two types of ultrasonic signals with a 300 V driving voltage at 300 KHz were used, as shown in Figure 3. The red and blue curves in the figure show the responses to the UW of the sensor based on matching FBGs and when using only one FBG; the two peak-to-peak values were 7.26 V and 4.08 V, respectively. The voltage of the ultrasonic signal detected by the sensor based on matching FBGs was nearly 1.8 times as high as the voltage based on one FBG. This is in accordance with the theoretical analysis and proves that our scheme is applicable in the real world. Additionally, the distance (2 mm) between the sensor and PZT source remained the same throughout the experiment.

**Figure 4:**
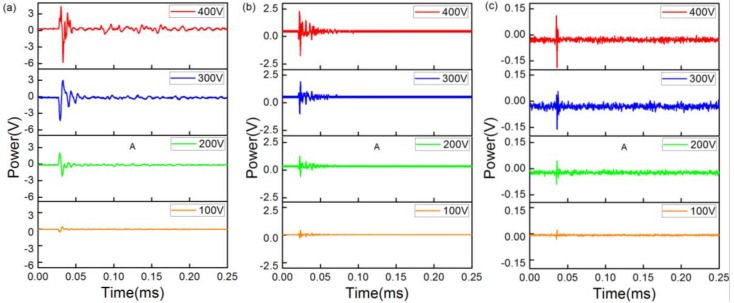
Responses of the sensor based on matching FBGs to a one-cycle ultrasonic pulse at (**a**) 300 KHz; (**b**) 1 MHz; and (**c**) 5 MHz.

The responses of the sensor using matched FBGs when using different UW frequencies were as follows. Figure 4a–c shows how the detection signals change with a decrease in the driving voltage at 300 KHz, 1 MHz, and 5 MHz, respectively. The sensor could detect UW with different driving voltages, as indicated by the data, which show decreased detection signals with a decrease in the driving voltage at the same frequency. Moreover, for the same driving voltage, there was a significant decrease as the ultrasonic frequency increased. This is due to the fact that the higher the acoustic frequency, the larger the transmission loss.

**Figure 5:**
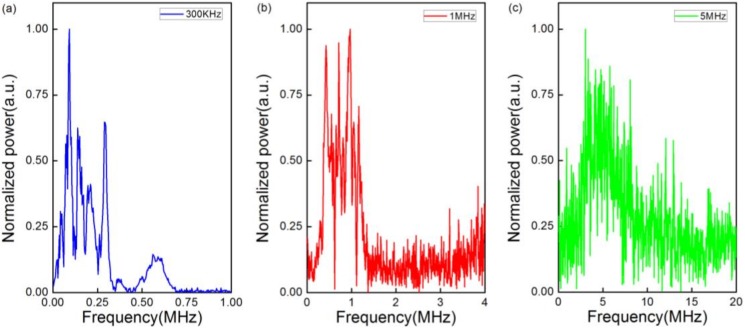
Frequency domain spectra of the ultrasonic pulse at (**a**) 300 KHz; (**b**) 1 MHz; and (**c**) 5 MHz.

The frequency domain characteristic of the signals is an important parameter of ultrasonic sensors. Figure 5a–c show the frequency domain of detected signals. It is obvious that, in Figure 5b,c, the center frequencies agree well with the acoustic source emissions . In fact, the center frequency in Figure 5a is also consistent with the acoustic source emission because the natural frequency of the PZT is 240 KHz rather than 300 KHz. Even though there are other frequencies near the center frequencies due to the inherent bandwidth of the PZT, the results indicate that the sensor is sensitive enough to the acoustic source emissions.

Another important parameter to estimate the performance of sensors is the SNR. The output signal was 56.9 dB at 300 KHz, 53 dB at 1 MHz, and 31.8 dB at 5 MHz. We can see that the SNR decreased as the frequency increased. This behavior is determined by the structure and the material of the sensor.

**Figure 6:**
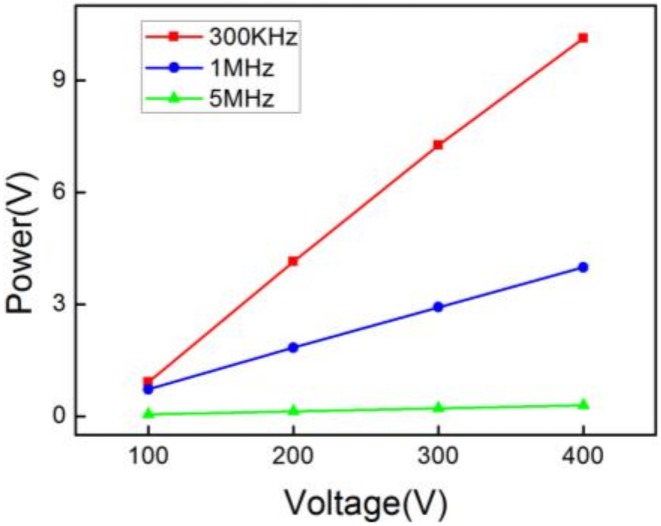
Pulsed ultrasound energy detection of 300 KHz, 1 MHz, and 5 MHz versus increasing voltage at a fixed distance.

To analyze the relation between the amplitude of detected signals and the driving voltage, we plotted the peak-to-peak acoustic voltages as a function of the driving voltage for each experimental input frequency. As shown in Figure 6, it can be seen that the peak-to-peak voltages are linear with the driving voltage. The slopes are 0.0307, 0.0109, and 0.0008, respectively. The detected acoustic voltage decreased with augmentation of the frequency, which shows that the sensor has excellent low-frequency characteristics.

**Figure 7:**
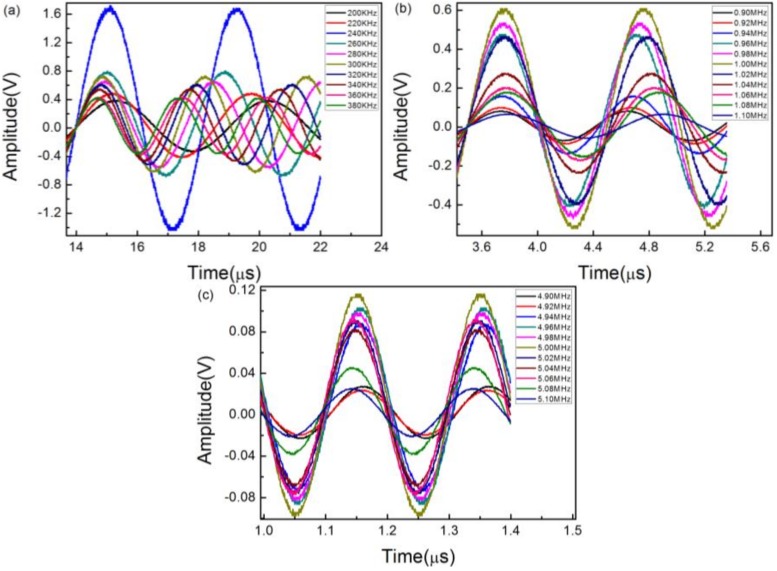
Sinusoidal ultrasonic signals at different frequencies using a piezoelectric transducer with a center frequency of (**a**) 300 KHz; (**b**) 1 MHz; and (**c**) 5 MHz.

We also detected the sinusoidal ultrasound at different frequencies to further verify the frequency characteristics of the sensor. In this process, three PZTs with center frequencies of 300 KHz, 1 MHz, and 5 MHz were employed for generating the ultrasonic signal. As shown in Figure 7a–c, no matter how the frequency changed, all the signals we detected are undistorted and distinguishable in the time domain.

**Figure 8:**
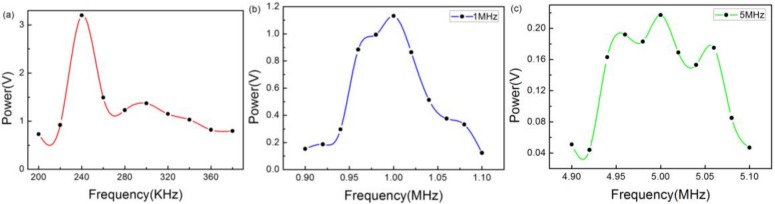
Amplitude-frequency response curves of the sinusoidal ultrasound using the PZT with a center frequency of (**a**) 300 KHz; (**b**) 1 MHz; and (**c**) 5 MHz.

However, their amplitudes varied with frequency. For the sake of further analysis on this phenomenon, amplitude–frequency figures were plotted, as shown in Figure 8a–c, which correspond to Figure 7a–c, respectively. As can be seen from Figure 7a–c, the maximum position corresponds exactly to the center frequency. Meanwhile, Figure 7a also verifies that the natural frequency of the PZT is 240 KHz. Additionally, the varying tendencies of the three curves are slight because the high-frequency performance of the sensor was not as strong as the low-frequency performance.

**Figure 9:**
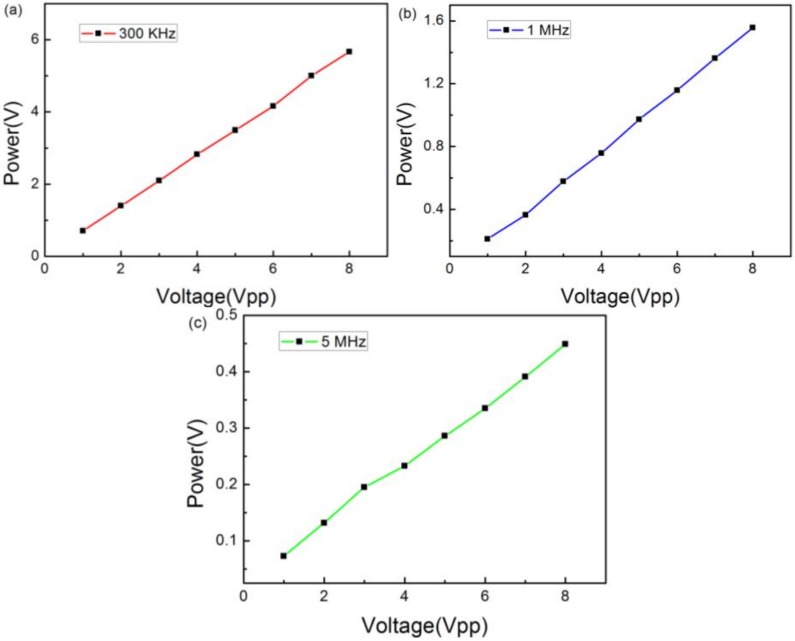
Sinusoidal ultrasound energy detection versus increasing voltage at a fixed distance at (**a**) 300 KHz; (**b**) 1 MHz; and (**c**) 5 MHz.

Figure 9a–c shows the detected acoustic voltages as a function of the driving voltage, which changes from 1 Vpp to 8 Vpp at 300 KHz, 1 MHz, and 5 MHz. We can see that the responses, similar to the ultrasonic pulse response, are linear. The gradients are 0.7044, 0.4025, and 0.0531, respectively. They differ from the ultrasonic pulse response because two types of function generators were employed to generate the signals. After further improvement, this sensor could be used to calibrate other sensors.

## 4. Conclusions

In this paper, we proposed a novel fiber optic sensor, based on a matched filtering method, to conduct acoustic measurements. The sensor consisted of two FBGs with extremely narrow spectra at different wavelengths. One of the FBGs was stretched and the other one was compressed under sound pressure. The synergic effect of the matching FBGs led the sensor to perform with high sensitivity to UWs. Side-band filtering technology-based intensity interrogation was adopted for acoustic detection. Additionally, the signal-to-noise ratios (SNR) of the output signals were 56.9 dB at 300 KHz, 53 dB at 1 MHz, and 31.8 dB at 5 MHz, respectively. Compared with other low-cost fiber optic ultrasonic sensors, it performed with a high SNR; the ultrasonic sinusoidal signals we detected were undistorted and distinguishable in the time domain, which is worthy of further study and could be used in the monitoring of maritime security.
